# Increased serum IL-6 is predictive of long-term cardiovascular events in high-risk patients submitted to coronary angiography: an observational study

**DOI:** 10.1186/s13098-022-00891-0

**Published:** 2022-08-26

**Authors:** Márcio Mossmann, Marco Vugman Wainstein, Stéfani Mariani, Guilherme Pinheiro Machado, Gustavo Neves de Araújo, Michael Andrades, Sandro Cadaval Gonçalves, Marcello Casaccia Bertoluci

**Affiliations:** 1 Post-Graduate Program in Medical Sciences: Cardiology and Cardiovascular Sciences, Universidade Federal do Rio Grande do Sul, Porto Alegre, Brazil; 2 Cardiology Division, Hospital de Clínicas de Porto Alegre, Porto Alegre, Brazil; 3 Endocrinology Division, Hospital de Clínicas de Porto Alegre (HCPA), Universidade Federal Do Rio Grande Do Sul (UFRGS), Ramiro Barcelos 2350, Porto Alegre, Rio Grande do Sul 90035-003 Brazil; 4 Internal Medicine Department, School of Medicine, UFRGS, Porto Alegre, Rio Grande do Sul Brazil; 5 Unidade de Análises Moleculares e de Proteínas (UAMP), Hospital de Clinicas de 17 Porto Alegre, Porto Alegre, Brazil

**Keywords:** Interleukin-6, Coronary artery disease, Diabetes, High-sensitive C-reactive protein, Inflammation

## Abstract

**Background:**

Interleukin-6 (IL-6) is an inflammation-related cytokine associated with an elevated risk of cardiovascular events. In a previous study, we demonstrated that increased IL-6 was predictive of sub-clinical atherosclerotic coronary disease in intermediate-risk patients undergoing coronary angiography. In the present study, we investigated whether increased serum IL-6 is predictive of cardiovascular events in high-risk patients.

**Methods:**

In this observational study, consecutive patients referred for elective coronary angiography due to stable chest pain/myocardial ischemia had IL-6 measured immediately before the procedure. Long-term follow-up was performed by phone call or e-mail, and their clinical registries were revised. The primary outcome was a composite of new myocardial infarction, new ischemic stroke, hospitalization due to heart failure, new coronary revascularization, cardiovascular death, and death due to all causes.

**Results:**

From 141 patients submitted to coronary angiography and IL-6 analysis, 100 had complete follow-up data for a mean of 5.7 years. The median age was 61.1 years, 44% were men, and 61% had type-2 diabetes. The median overall time-to-event for the primary outcome was 297 weeks (95% confidence interval [CI] 266.95–327.16). A receiver operator characteristic curve defined the best cut-off value of baseline serum IL-6 (0.44 pg/mL) with sensitivity (84.37%) and specificity (38.24%) to define two groups. High (> 0.44 pg/mL) IL-6 levels were predictive of cardiovascular events. (*p* for interaction = 0.015) (hazard ratio = 2.81; 95% CI 1.38–5.72, p = 0.01). Subgroup analysis did not find interactions between patients with or without diabetes, obesity, or hypertension.

**Conclusion:**

In conclusion, an interleukin-6 level higher than 0.44 pg/mL, obtained just before elective coronary angiography, was associated with a poorer prognosis after a mean of 5,7-year. A pre-procedure IL-6 below 0.44 pg/mL, on the other hand, has a very good negative predictive value, suggesting a good prognosis, and may be useful to better indicate coronary angiography in high-risk patients.

.

## Background

Interleukin-6 (IL-6) is an acute-phase protein that plays a significant role in the inflammatory response, vascular inflammation, and atherosclerosis process [1]. It contributes to the remodeling of connective tissue by increasing metalloproteinase gene expression [2]. Focal overexpression of activated metalloproteinase may promote destabilization and degradation of the plaque’s fibrous cap, leading to plaque instability during the atherosclerotic process [3]. In a study including patients with unstable coronary artery disease (CAD), very high IL-6 levels (> 5 pg/mL) were strongly associated with mortality, which was independent of many risk factors, including age, sex, diabetes, previous myocardial infarction (MI), and high cholesterol levels [4]. In a nested case–control study of patients with previous MI, the risk of future MI increased progressively with increasing quartiles of baseline IL-6 concentration [5].

IL-6 is also predictive of cardiovascular events in patients with stable coronary disease. In a sub-study from the *Stabilization of Atherosclerotic Plaque by Initiation of Darapladib Therapy Trial* (STABILITY), higher levels of IL-6 were independently associated with the risk of major adverse cardiovascular events, cardiovascular and all-cause mortality, MI, heart failure, and cancer mortality [6]. Recently, our group demonstrated an association between serum IL-6 concentrations and subclinical CAD, in which high levels of serum IL-6 level (> 1 pg/mL) were predictive of coronary stenosis ≥ 30% in intermediate-risk patients referred for coronary angiography [7].

Although there is a growing body of evidence associating IL-6 with cardiovascular disease, most are indirect observations from case–control studies or sub-group analyses. IL-6 has been inadequately studied prospectively concerning its predictive value. Much less is known about the role of IL-6 in patients referred for elective coronary angiography. Moreover, obesity and non-cardiovascular inflammatory diseases considerably interfere with serum IL-6 levels, which may lead to a confusing bias. In the present study, we aimed to prospectively analyze the impact of higher and lower levels of IL-6 on cardiovascular events in patients with high or very high cardiovascular risk, excluding severely obese patients and patients with previously known inflammatory conditions.

## Methods

### Study design

This is an observational study divided into two phases: an initial cross-sectional phase and an observational prospective cohort phase. The inclusion period was from October 2012 to August 2016. We screened potential participants who were referred to the cardiology division catheterization laboratory (Cath Lab) of Hospital de Clínicas, a large tertiary care university hospital in southern Brazil.

**Fig. 1:**
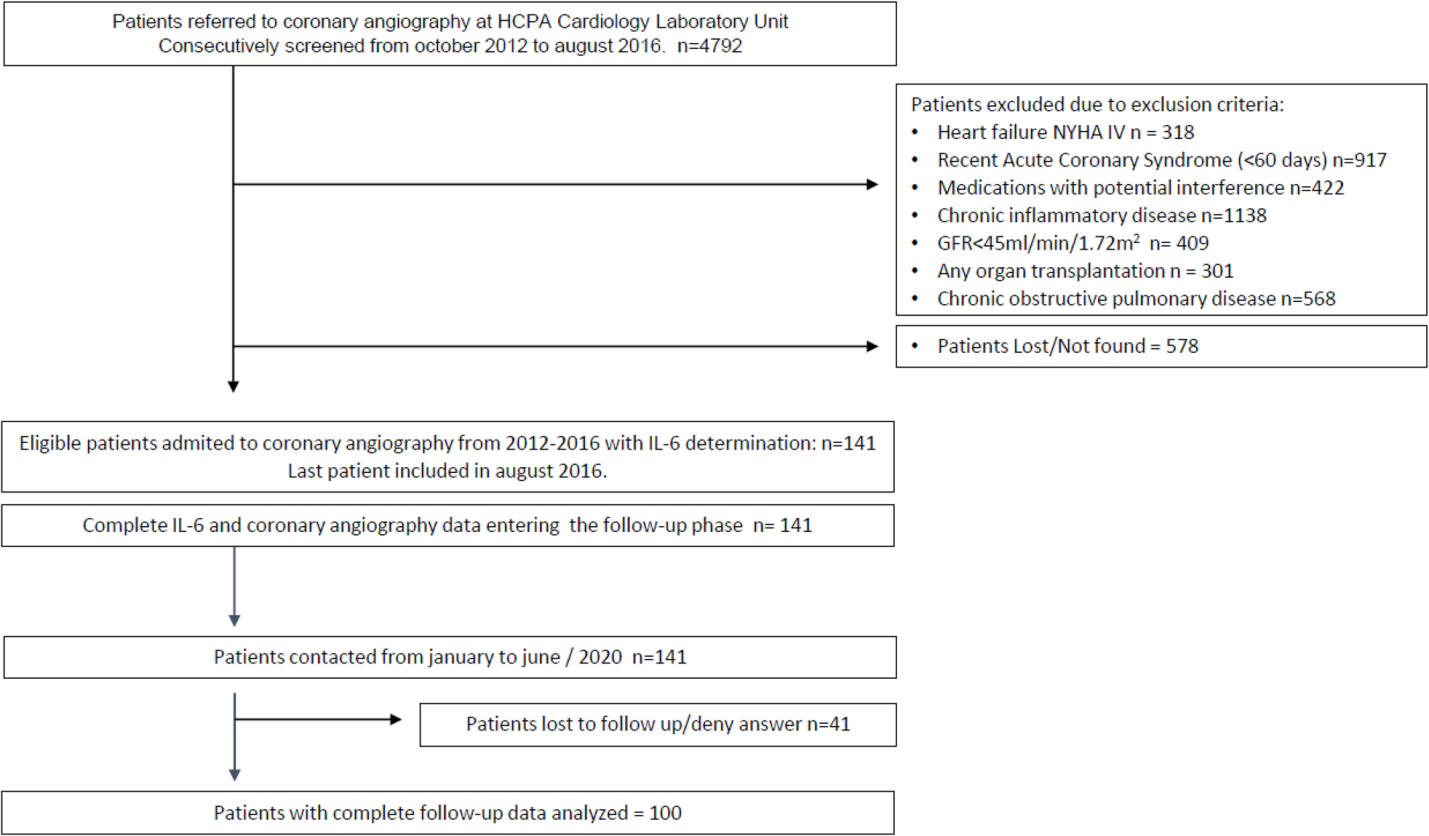
Flowchart of the inclusion process

We considered for inclusion every patient referred for elective coronary angiography due to non-acute chest pain or chronic myocardial ischemia confirmed by non-invasive investigation and who did not have any exclusion criteria (Fig. 1).

Inclusion and exclusion criteria were checked immediately before the procedure by the investigators through a hospital registry review and direct personal interview. If the patients were eligible and agreed to participate in the study, a signed consent, anthropometric data, and a fasting blood sample for IL-6 and blood chemistry were obtained, and blood pressure was measured in the sitting position. This study was approved by the Institutional Research Ethics Committee (IRB number 18-0221).

After the procedure, patients were discharged from the unit and were referred to their respective assistant physicians. From March 2020 to August 2020, all patients were contacted by one of the investigators through multiple phone calls and e-mail, and their hospital and city obituary registries were reviewed.to obtain the most recent clinical information available.

### Inclusion and exclusion criteria

We selected patients between the ages of 30–80 years with were suspected of CAD due to a history of chronic chest pain or stable myocardial ischemia confirmed through a non-invasive test. Considering current guidelines and clinical trials [8], in our practice, we indicated coronary angiography and stenting for patients with large areas of ischemia; left main disease on coronary-CT; reduced ejection fraction, and/or three-vessels disease. We considered high-risk patients defined by the presence of at least 3 traditional risk factors, or the presence of sub-clinical atherosclerosis (less than 50% of stenosis) presented at coronary angiography [9]. Patients with previous myocardial infarction events, coronary revascularization, or more than 50% coronary stenosis were considered at very high risk.

We excluded patients with known congestive heart failure, class-IV New York Heart Association, recent acute coronary syndrome, defined as any confirmed acute coronary syndrome occurring in coronary in the last 60 days; clinically significant renal disease defined as a glomerular filtration rate of less than 45 mL/min/1.73 m^2^; any known inflammatory conditions such as chronic pulmonary obstructive disease confirmed by thorax X-Ray and spirometry; known active chronic infectious diseases such as tuberculosis (defined by typical lesion on thorax X-ray), HIV infection (detected by antibody against HIV), chronic rheumatic disease (rheumatoid arthritis and systemic erythematous lupus detected by antinuclear antibodies); chronic B or C hepatitis (detected by HBsAg and HCV antibodies), hyper or hypothyroidism determined by a low or high TSH (less than 0.01mUI/L or above 5 mUI/L), a history of any organ transplantation or patients undergoing evaluation for transplantation, and any known type of cancer. We also excluded severely obese patients with a body mass index (BMI) > 35 kg/m^2^ and those taking medications that might interfere with the inflammatory status of the patient, such as corticosteroids, HIV-antiretroviral, carbamazepine, phenytoin, any drug for cancer, immunosuppressant, nitrofurantoin, anti-malaria, lithium, and anti-psychotic drugs. We did not exclude patients with diabetes or hypertension.

### Coronary artery angiography procedure

Coronary angiography was always performed in the morning, in the fasting state. We used the Axiom Artis Siemens^®^ equipment (Germany) in all patients. Two experienced interventional cardiologists, who were blinded to all other clinical variables, made all the angiographic measurements. Angiographic analyses were made by visual (non-quantitative) estimates of luminal narrowing in at least two different orthogonal projections. The presence of CAD was defined as any lesion causing > 30% reduction in the diameter of an epicardial coronary artery.

### Clinical and biochemical investigation

#### Blood pressure measures

Baseline blood pressure was measured at the Cath Lab before coronary angiography, after 15 min of rest, in the sitting position, in the right arm. Three sequential measurements were made using an automatic aneroid sphygmomanometer (OMRON Comfort III Visomat Incoterm, Germany). We considered the lowest blood pressure reading as the final measure.

#### IL-6 measures

Blood samples were collected at the Cath Lab just before the beginning of coronary angiography. For serum IL-6 measurement, a custom Luminex® assay was employed (Invitrogen®, #LHB0001CM) following the manufacturer’s instructions. Briefly, 50 μL of the undiluted sample was added to wells containing buffers and magnetic beads. After 2 h of incubation (550 rpm), the wells were washed and the detection antibody was added further for 1 h. After washing, streptavidin–phycoerythrin was added further for 30 min, the wells were washed again, and the beads were suspended in 125 μL of wash buffer. Beads were read in Luminex^®^ x-Map 200, and a minimum of 100 events was recorded for each bead. The limit of detection was defined as the lowest standard value (0.08, pg/mL). Values are expressed as pg/mL.

#### Other assays

Blood samples for high-sensitivity C-reactive protein (hs-CRP) were also collected simultaneously and aliquoted. Serum hs-CRP levels were determined using the turbidimetric immunoassay method (Roche^®^). Serum creatinine (Jaffé method), lipid profile, glycated hemoglobin (high-performance liquid exchange chromatography), and glucose (colorimetric assay) were also measured.

### Follow-up phase

After discharge, all included patients were contacted through phone calls by one of the investigators from March to August 2020, following a specific protocol. Patients were required to confirm their clinical outcomes through medical registries. In-hospital registries were also obtained from those who continued to visit the hospital. Information regarding death was confirmed by a family member who attended the call, and their city obituary data were confirmed. Patients who could not be contacted after several attempts and had no further clinical hospital registry information after discharge were considered missing at random.

### Outcomes

We analyzed a composite of six cardiovascular outcomes: (1) new acute coronary syndrome or MI, according to the Universal Definition of Myocardial Infarction updated, including unstable angina; (2) hospitalization due to heart failure, with elevated levels of B-type natriuretic peptide (BNP) and/or NYHA class of symptoms III or IV; (3) hospitalization due to ischemic stroke, with new-onset neurological deficit with corresponding imaging lesion on CT or MRI scan; (4) new coronary revascularization, new symptoms with positive non-invasive ischemia assessment; (5) cardiovascular death, and (6) death due to all causes. Only the first event after the coronary angiography was considered.

Time-to-event was expressed in weeks and confirmed using medical records and phone calls to the patients. The follow-up period was defined as the period between the date of discharge from the Cath Lab and the date of the first outcome reported or the last contact if no outcomes occurred.

### Statistical analysis

Continuous variables were expressed as mean ± standard deviation [SD]) or median (interquartile range) based on their symmetrical or asymmetrical distribution, respectively. The normality of the distribution of each variable was assessed using the Shapiro–Wilk test. Categorical variables were represented by their relative and absolute frequencies.

Patients were divided according to their baseline IL-6 levels into 2 groups—above and below 0.44 pg/mL. This cut-off value was chosen from the ROC curve analysis as the best cut-off point for sensitivity and specificity. Patient groups were compared using the independent samples Student’s t-test or Kruskal–Wallis test, as appropriate, for continuous variables and Fisher’s exact tests for categorical variables. The Kaplan–Meier analyses and comparison using the log-rank test were performed using MedCalc Statistical Software version 14.8.1 (MedCalc Software bvba, Ostend, Belgium). All remaining statistical analyses were conducted using SPSS Statistics for Windows, Version 21.0. (IBM Corp., Armonk, NY, USA).

The sample size was based on a previous paper from our group [7] in which we compared IL-6 in individuals with and without coronary artery disease. In that study, we observed IL-6 serum levels of 1.37 pg/mL in the group with coronary artery disease and IL-6 of 0.29 pg/mL in the non-CAD group, a difference of 1.08. In the present study, as all patients had coronary artery disease we arbitrarily decided to consider a smaller difference between groups (0.5 pg/mL). To accept an alpha error of 0.05 we estimated a sample size of 100 patients, which would generate a power of 0.73.

All patients provided written informed consent (the ethics committee of the Hospital de Clínicas approved the study protocol).

## Results

A detailed flowchart of the inclusion process is depicted in Fig. 1. A total of 4792 cardiac catheterizations were performed at the Cath Lab between October 2012 and August 2016. During this period, 141 patients were selected according to the inclusion and exclusion criteria. Of these, 100 were analyzed with complete follow-up data.

Baseline clinical and anthropometric characteristics are shown in Table 1. Forty-four percent were men and the median age of all patients was 61.1 years. CAD, that is > 30% stenosis, was present in 81% of the patients and was similar between both sub-groups of IL-6. No patient had left main coronary disease. There were a higher proportion of patients with more severe coronary disease, including two-vessel and multivessel disease, in the higher IL-6 group. The proportion of sub-clinical coronary atherosclerosis was similar between both IL-6 groups. There was also a higher proportion of patients with hypertension and type-2 diabetes (T2DM) and a trend toward a higher proportion of obese patients in the group with higher levels of IL-6. The mean hs-CRP level, as expected, was increased in the group with higher levels of IL-6.

**Table 1:** Overall baseline characteristics according to IL-6 group

	Overall	IL-6 ≤ 0.45	IL-6 > 0.45	*p*
n	100	32	68	
Age (years)	61.1 (8.7)	58.4 (7.1)	62.8 (9.2)	
Male	44 (44.0)	11 (34.4)	34 (50.0)	0.196
BMI (kg/m^2^)	28.7(5.6)	27.1 (5.4)	29.5 (5.6)	0.053
AC (cm)	100.7 (14.3)	95.7 (12.1)	103.1 (14.7)	0.015
SBP (mmHg)	140(22)	136.4 (20.7)	142.1(23.2)	0.236
DBP (mmHg)	76.9 (12.6)	75.1 (10.40)	77.8 (13.5)	0.328
% of hypertension	85 (85.0)	22(68.8)	63 (92.6)	0.005
CAD > 30%	81 (81.0)	23 (71.9)	58 (85.3)	0.170
n of T2DM (%)	61 (61.0)	10 (31.3)	51 (75.0)	< 0.001
n of current smoking (%)	20 (20.0)	5(15.6)	15(22.1)	0.595
n with decreased eGFR (%)	45 (45.0)	11 (34.4)	34 (50.0)	0.196
n with previous AMI (%)	28 (28.0)	6 (19.4)	22(32.8)	0.231
n with heart failure (%)	17 (17.0)	2 (18.2)	15 (29.4)	0.712
HbA1c (%)	6.8 (1.50)	6.3 (1.4)	7.1 (1.5)	0.02
Blood glucose (mg/dL)	112 (92–154)	97 (88 – 127)	117 (99 – 164)	0.016
Total cholesterol (mg/dL)	163 (133.5–194.5)	179 (134–200)	158 (132 – 182)	0.075
Serum creatinine (mg/dL)	0.82 (0.2)	0.72 (1.5)	0.86 (0.2)	0.001
IL-6 (pg/mL)	1.10 (1.6–2.10)	0.09 (0.04–0.14)	1.5(0.96–2.40)	< 0.001
hs-CRP	2.6 (1.2 -7.9)	1.63 (1.0 -2.8)	3.9 (1.5–8.7)	0.008
% Using aspirin	71.0	67..7	74.6	0.478
% Using clopidogrel	16.0	27.3	25.5	1.000
% Using ACEi	35.0	54.5	56.9	1.000
% Using ARB	19.0	18.2	33.3	0.478
% Using beta blockers	48.0	72.7	78.4	0.700
% Using statins	83.0	80.6	86.6	0.548

Values are expressed as mean** ± **standard deviation, median with interquartile range or proportion (%)

BMI, body mass index; AC, Abdominal circumference; SBP, systolic blood pressure; DBP, diastolic blood pressure; CAD, Coronary Artery Disease; T2DM, type 2 diabetes mellitus; eGFR, estimated glomerular filtration Rate (CKD-EPI formula); AMI, acute myocardial infarction; HF, heart failure; HbA1c, Hemoglobin A1c; IL-6 interleukin-6. hs-CRP high-sensitive C-reactive protein; ACEi, angiotensin-converting enzyme inhibitors; ARB, angiotensin II receptor blocker

**Fig. 2:**
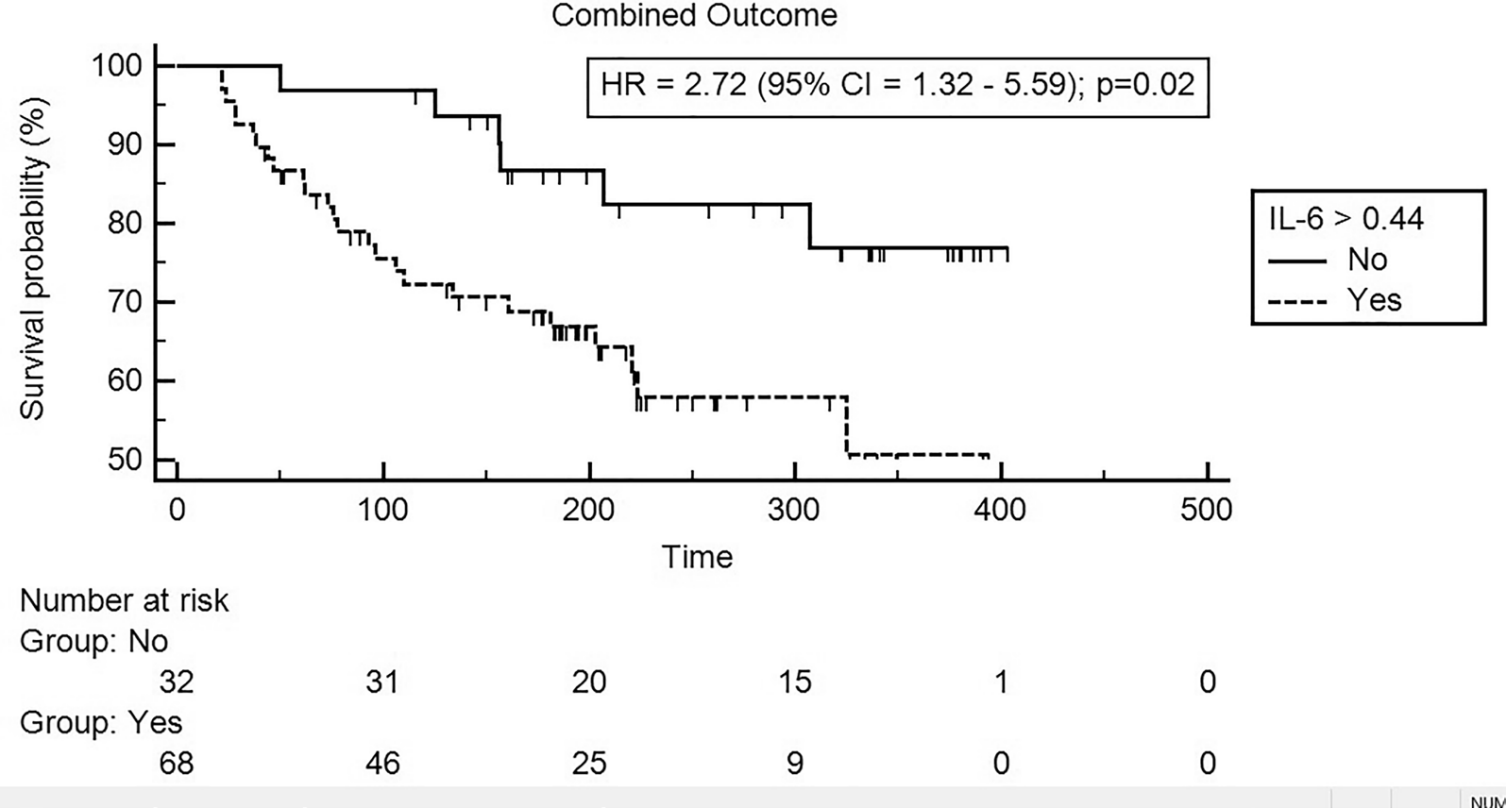
Time-to-event curves for composite outcome according to IL-6 levels. Event rates are calculated with the use of Kaplan-Meier methods and compared with the use of the log-rank test. *IL-6* interleukin-6

The median overall time-to-event for the primary outcome was 297 weeks (95% confidence interval [CI] 266.95–327.16). The outcomes are shown in detail in Table 2. Overall, 32 cardiovascular events occurred during the follow-up period, being 26 in the higher IL-6 group and 6 in the lower IL-6 group. Figure 2 shows the Kaplan–Meier curves with the hazard ratios (HRs) of primary outcome between high and low IL-6 groups. There was a significantly higher cumulative incidence of the primary outcome during the follow-up period in the group of patients with increased baseline IL-6 (HR = 2.81; 95% CI 1.38. 5.72, *p* for interaction = 0.015).

**Table 2:** Cardiovascular events and mortality outcomes

Outcome	Overall	Il-6 ≤ 0.44	IL-6 > 0.44	*p*-value
All combined (%)	32 (32.0)	6(18.8)	26 (38.2)	0.06
Acute myocardial infarction (%)	1 (1.0)	0 (0.0)	1 (1.5)	1.000
Unstable Angina (%)	8 (8.0)	2 (6.3)	6 (8.8)	1.000
Heart failure hospitalization (%)	10 (10.0)	1 (3.1)	9 (13.2)	0.162
Ischemic stroke	0	0	0	1.000
CV death (%)	2 (2.0)	0 (0.0)	2 (2.9)	1.000
New re-vascularization (%)	10 (10.0)	3 (9.4)	7 (10.3)	1.000
All-cause mortality (%)	1 (1.0)	0 (0.0)	1 (1.5)	1.000

Data are the total number of events and the percent within each group

**Fig. 3:**
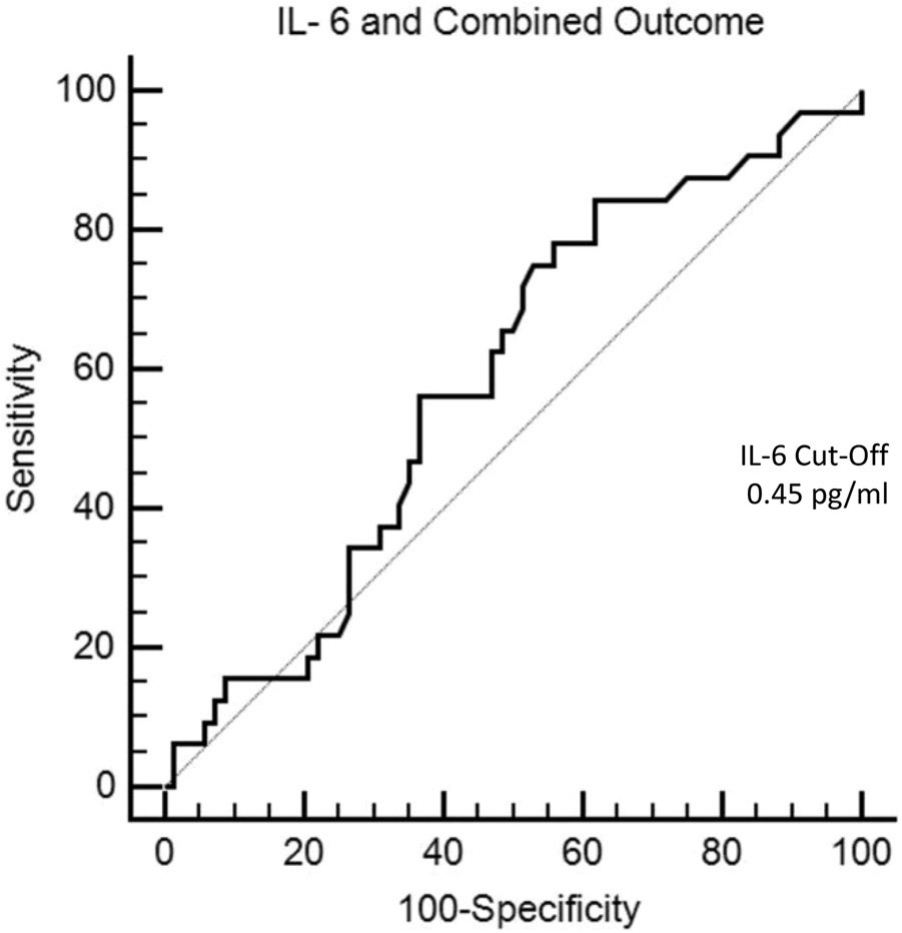
Receiver operator characteristic (ROC) graph showing areas under the curve IL-6 for the composite outcome IL-6: interleukin-6

The area under the ROC curve of IL-6 for the combined outcome was 0.585 (95% CI 0.482–0.683; *p* = 0.156; Fig. 3) with a sensitivity of 81.25 (95% CI 63.6– 92.8), specificity of 38.24 (95% CI 26.7–50.8), positive predictive value of 38.2 (95% CI 26.7–50.8), negative predictive value of 81.2 (95% CI 63.6–92.8) and a power of 0.73.

To examine if IL-6 correlates with cardiovascular risk, we considered hypertension, type 2 diabetes, smoking, male gender, age above 60 years, and the presence of CHD as isolated risk factors and correlated with IL-6. As IL-6 has a skewed distribution we used the Spearman correlation test. We found a Spearman rho of 0.428 with a p-value < 0.0001, indicating a definite correlation between cardiovascular risk and IL-6 levels here (data not shown).

**Fig. 4:**
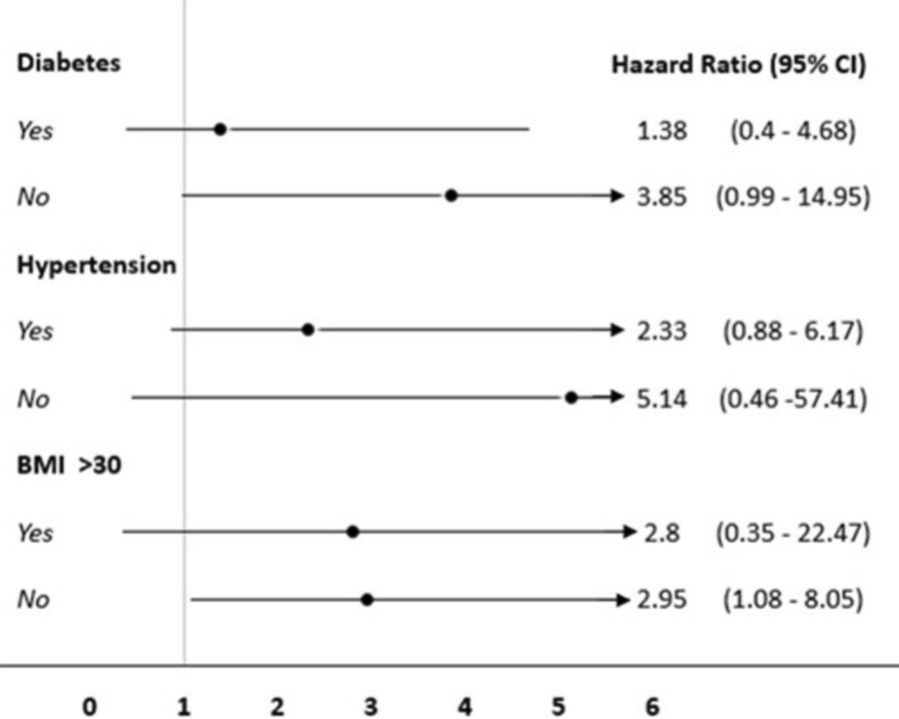
Forrest plot of subgroup analysis for the presence or absence of T2DM, hypertension, and obesity T2DM: type-2 diabetes mellitus

Although the proportion of patients with T2DM was higher in the group with higher IL-6 levels (Table 1), a sub-group analysis between patients with and without T2DM examining the presence or absence of the primary outcome (Fig. 4) did not show any interaction. Moreover, The subgroups analysis of patients with and without hypertension and with or without BMI > 30 kg/m^2^, regarding cardiovascular outcomes, did not show interaction as well.

## Discussion

The present study shows that serum IL-6 is predictive of long-term cardiovascular events in symptomatic patients with stable coronary disease who have a high cardiovascular risk. Serum IL-6 measurements above 0.44 pg/mL increased the risk of cardiovascular events by 2.8 times. Although there was an increased proportion of T2DM, hypertension, and obesity in the group with increased levels of IL-6, there was no interaction among these variables considering the primary outcome. Although the correlation between IL-6 and coronary events is widely known, this study highlights its strong negative predictive value, with potential clinical application in indicating coronary angiography in high-risk patients.

A previous study based on the analysis of two population-based cohorts [10] suggested that circulating serum IL-6 levels could be associated with increased coronary risk (defined as nonfatal MI or fatal coronary heart disease [CHD]). In that study, stored blood samples of patients who later developed non-fatal MI or died of CHD were used for baseline measurements. Patients who developed CHD had greater levels of IL-6 compared with controls with no history of CHD. The odds ratio for CHD, adjusted for several established risk factors, was 1.46 (95% CI 1.29–1.65) per 2 SDs of increase in baseline IL-6 values.

IL-6 has been associated with increased cardiovascular risk in some populations. In a meta-analysis of 29 population-based prospective studies [11], the adjusted relative risk for non-fatal MI or CHD death was 1.25 for every point of higher baseline SD in IL-6. However, in this meta-analysis, there was considerable heterogeneity (*I*^2^ = 53.6%, *p* = 0.001), and not all studies included indicated a clear risk prediction for IL-6. One possible reason is that many co-variables may have impacted the results in some studies.

In the present study, we observed that the predictive cut-off value of IL-6 was relatively lower (0.44 pg/mL) compared to that in other studies. In the sub-analysis of the STABILITY trial [6], the risk of cardiovascular death and major adverse cardiovascular events in 3–4 years started to increase progressively when IL-6 levels were above 1.5 ng/L. In the FRISC II trial [4], the highest predictive value was obtained when IL-6 levels were above > 5 pg/mL. We attribute our findings to the fact that we were able to exclude patients with chronic inflammatory diseases and severe obesity, which are major confounders when studying sub-clinical vascular inflammation as there is a strong relationship *(rho* = 0.85; *p* < 0.00001) between IL-6 levels and BMI [12]. We believe that the strict selection criteria improved the accuracy of IL-6, also increasing the negative predictive value.

There is a clear plausibility for IL-6 levels to be associated with a higher risk of cardiovascular events. Experimental studies indicate that vascular endothelial and smooth muscle cells from normal and aneurysmal arteries can produce IL-6 [13, 14]. Moreover, IL-6 gene transcripts are expressed in atherosclerotic lesions [15], confirming local production. IL-6 has procoagulant effects [16], and elevated levels have been reported among patients with acute coronary syndromes [17]. Considering that atherosclerosis is a chronic inflammatory disorder, IL-6 levels are expected to be increased among individuals with sub-clinical atherosclerosis who are at greater risk for future MI. However, a cause-and-effect relationship between IL-6 and cardiovascular events cannot be clearly defined so far, as few randomized trials are targeting IL-6 treatment.

In the *Canakinumab Anti-inflammatory Thrombosis Outcome Study* (CANTOS), a randomized, double-blind, placebo-controlled trial involving stable patients with previous myocardial infarction, the human monoclonal antibody, canakinumab, that targets the interleukin-1β innate immunity pathway, led to a significant decrease of recurrent cardiovascular events compared to placebo, independently of lipid-level lowering [18]. In this trial, patients had a clinical atherosclerotic disease and a high mean baseline IL-6 (2.54 pg/mL), consistent with an inflammatory state. After 48 months, canakinumab 150 mg reduced significantly the relative event rate by 15%. In a sub-analysis of the CANTOS trial [19] patients who achieved IL-6 levels lower than 1.65 pg/mL experienced a 32% reduction in cardiovascular events, a 52% reduction in cardiovascular mortality, and a 48% reduction in all-cause mortality. These results suggest the existence of a clear association between lower levels of IL-6 and reduced incidence of cardiovascular events.

Despite the substantial IL-6 fall seen in the CANTOS study, the mean IL-6 levels at 3 months seen in that study, continued high even in the group with the lowest IL-6 (1.93 (1.45–2.63 pg/mL). The mean IL-6 achieved in the present study, however, was much lower than in CANTOS. In our study, the mean IL-6 in the group with low levels was 0.09 pg/mL (95% CI 0.04–0.14) and was associated with a low incidence of cardiovascular events.

In the present study, because we selected patients excluding the most common inflammatory confounders that could raise IL-6, we were able to include patients with even normal levels of IL-6, despite the established coronary disease. This allowed us to evaluate the negative predictive value of IL-6. We observed that levels of IL-6 below 0.44 pg/mL were predictive of a better prognosis in 82%, an indication of a lower inflammatory status seen in that group. This may be a useful tool to evaluate the cardiovascular risk of candidates for coronary angiography.

The first limitation of this study is the sample size. Thus, the borderline difference in combined outcomes found between groups may be subject to beta error. Yet, the high-risk patients and long follow-up with a considerable incidence of combined outcomes may have balanced the small sample size. Second, as an observational study, we could not confirm a cause-effect relationship between IL-6 levels and the incidence of cardiovascular events. It is also possible that IL-6 may be a marker of cardiovascular disease rather than a risk factor per se. However, the study confirms the strong body of literature on this subject and highlights IL-6 applicability in a specific context. Third, we were not able to make adjustments to covariates due to the relatively low incidence of cardiovascular events. Nonetheless, this is a sample of patients at high cardiovascular risk that frequently has many comorbidities.

## Conclusions

In conclusion, levels of interleukin-6 higher than 0.44 pg/mL obtained just before the coronary angiography for elective reasons were associated with a poorer prognosis after a mean of 5.7-year. A pre-procedure IL-6 below 0.44 pg/mL, on the other hand, has a very good negative predictive value, indicates a good prognosis, and may be useful to better indicate coronary angiography in high-risk patients.

## Data Availability

The datasets during and/or analyzed during the current study are available from the corresponding author on reasonable request.

## Abbreviations

CAD: Coronary artery disease
IL-6: Interleukin-6
MI: Myocardial infarction
Cath Lab: Catheterization laboratory
T2DM: Type-2 diabetes mellitus
CI: Confidence interval
HR: Hazard ratio
CHD: Coronary heart disease
SD: Standard deviation
ROC: Receiver operator characteristic
CT: Computed tomography
MRI: Magnetic resonance imaging
CKD: Chronic kidney disease
